# Is hepatic resection always a better choice than radiofrequency ablation for solitary hepatocellular carcinoma regardless of age and tumor size?

**DOI:** 10.17305/bb.2022.8507

**Published:** 2023-08-01

**Authors:** Shicong Zeng, Yao Zhang, Zongwen Wang, Xiaohang Ren, Jingtao Li, Shuoheng Ma, Wenyu Liu, Qiankun Zhu, Yan Yan, Bo Zhai

**Affiliations:** 1 Department of Surgical Oncology and Hepatobiliary Surgery, The Fourth Affiliated Hospital of Harbin Medical University, Harbin, Heilongjiang, China; 2 Department of Intervention Radiology, The Fourth Affiliated Hospital of Harbin Medical University, Harbin, Heilongjiang, China

**Keywords:** Hepatocellular carcinoma (HCC), hepatic resection (HR), radiofrequency ablation (RFA), tumor size, elderly, Surveillance, Epidemiology, and End Results (SEER)

## Abstract

In this study, we aimed to compare survival outcomes after receiving radiofrequency ablation (RFA) and hepatic resection (HR) for solitary hepatocellular carcinoma (HCC) with stratification by tumor size and age. A retrospective cohort was obtained from the Surveillance, Epidemiology, and End Results (SEER) database from 2004 to 2015. Patients were grouped by tumor size (0–2, 2–5, and >5 cm) and age (>65 and ≤65). Overall survival (OS) and disease-specific survival (DSS) were assessed. For patients >65 with tumors measuring 0–2 and 2–5 cm, the HR group had better OS and DSS compared with the RFA group. For patients >65 with tumors >5 cm, OS and DSS did not differ significantly between the RFA and HR groups (*p* ═ 0.262 and *p* ═ 0.129, respectively). For patients ≤65, the HR group had better OS and DSS compared with the RFA group regardless of tumor size. For patients with resectable solitary HCC, regardless of age, HR is the better choice not only for tumors ≤2 cm but also for tumors 2–5 cm. For resectable solitary HCC with tumors >5 cm, HR is the better choice for patients ≤65 but for patients >65, the issue of treatment choice needs to be further studied.

## Introduction

According to the global cancer report from the International Agency for Research on Cancer in 2020, liver cancer is the third most common cause of cancer-related death and the sixth most common cancer worldwide [1]. Liver cancer incidence and mortality have been increasing in many areas of the world and declining in some Asian countries in a recent report [2–4]. Hepatocellular carcinoma (HCC) is the dominant histologic type of primary liver cancer, accounting for approximately 90% of total cases [5]. Numerous risk factors, including hepatitis B virus (HBV) and hepatitis C virus (HCV) infection, excessive alcohol consumption, aflatoxin exposure, smoking, and obesity, are responsible for the development of HCC [6]. The aggressive behavior of malignancy and insufficient early diagnostic precision contributes to the poor prognosis and high mortality of HCC.

Currently, the Barcelona Clinic for Liver Cancer (BCLC) staging system is most frequently used to offer prognostic information and treatment recommendations [7, 8]. According to the BCLC staging system, early-stage HCC is defined as a solitary lesion irrespective of tumor size or no more than three tumors each <3 cm in size (without vascular invasion or extrahepatic spread) with preserved liver function and resection, ablation, or transplantation are recommended as the three preferred treatments for these patients [9]. For patients with solitary resectable HCC and well-preserved liver function, hepatic resection (HR) is the first-line treatment option, with 5-year survival rates of over 70% [10]. Radiofrequency ablation (RFA) is recommended for early multifocal HCC (each no more than 3 cm) and for single, small HCC with dissatisfactory liver function. Bioeffects of RFA are based on alternating electrical current (300–1000 kHz) through an electrode tip inserted into the HCC that induces heat reaching temperature of 60 ^∘^C–100 ^∘^C, which leads to coagulative necrosis of tissue [11]. Currently, RFA is the most widely adopted ablation technique because it can achieve complete response rates in about 90%–100% of HCC lesions below 3 cm, with the ability to obtain clear surgical margins. For patients meeting the Milan criteria, defined as a single lesion smaller than 5 cm or 2–3 lesions no larger than 3 cm and no macrovascular invasion, liver transplantation (LT) is a better choice compared with RFA and HR, as RFA and HR are hampered by high risk of HCC recurrence [12–14]. Globally, a shortage of donor livers evolved as the main restriction of LT to some extent [15, 16]. Therefore, RFA and HR are the commonly performed treatments for solitary HCC.

Numerous prognostic factors of HCC have been identified, such as tumor size, tumor lesion, and age [17–19]. Several studies have compared the efficacy of RFA and HR in HCC patients with different-sized tumors but failed to draw consistent conclusions. For solitary HCC with tumors ≤2 cm, some studies pointed out that although ablation and resection have similar survival outcomes, RFA rather than HR should be performed [20–22]. Nevertheless, a study conducted by Liu et al. reported that HR was preferred over RFA in this population [23]. For a single tumor measuring 0–5 cm, Chen et al. concluded that HR provided similar overall survival (OS) and disease-free survival (DFS) compared with RFA; however, ablation showed an advantage over surgical resection in causing less post-treatment complications, less pain, and a shorter in-hospital stay [24]. Salhab and Canelo [25] suggested that RFA was not applicable as a first-line treatment in patients with a single small HCC tumor >3 cm because when tumors are greater than 3 cm, RFA is characterized by high incomplete ablation and local recurrence rates. Tumor size larger than 5 cm is regarded as a contradiction for HR because a study revealed that the cancer-specific survival of patients with tumors >5 cm was significantly worse than for those with tumors ≤5 cm and the proportion of intrahepatic recurrences in the >5 cm group were approximately 1.4 folder higher than that in the ≤5 cm group [26].

Another important prognostic factor is age. In recent years, the amount of elderly HCC patients has gradually increased with the improvement of medical care [27, 28]. It is reported that more than 50% of hepatic malignancy occur in patients above 65 years [29]. Elderly patients often carry more comorbidities, altered physiology, and pharmacokinetics [30]. Therefore, treatment options for these patients may be different from those of younger individuals. Up to now, few studies have compared the efficacy of RFA and HR in elderly patients with HCC. Hence, this study was designed to evaluate the efficacy of HR and RFA as primary treatment for solitary HCC patients with stratification by age and tumor size.

Data regarding treatment and outcomes of solitary HCC patients in the Surveillance, Epidemiology, and End Results (SEER) database were examined. For the first time, we compared the efficacy between HR and RFA in patients older than 65 compared with those younger than 65 with a large sample size. In order to explore the influence of age and tumor size on the clinical decision making of solitary HCC patients, we conducted a subgroup analysis to determine whether resection or ablation is the better choice. After propensity score matching (PSM) with a large sample, OS and disease-specific survival (DSS) were compared between patients who underwent HR and patients who underwent RFA.

## Materials and methods

### Patient population

Data were extracted from the SEER database using SEER*Stat software version 8.3.9. In this study, a total of 47,799 patients diagnosed with HCC in the SEER database from 2004 to 2015 were identified. The inclusion criteria were as follows: (1) the primary site of the tumor was the liver (C22.0); (2) the histologic type was HCC (ICD-0-3: 8170–8175); (3) liver cancer was the first primary tumor (tumor sequence number: one primary only or 1st of 2 or more primaries); (4) histopathology confirmed (diagnosis confirmation: positive histology); (5) HR (SEER code: 20–26, 30, 36–38, 50–52, 59, 60, 66, 90) or RFA (SEER code: 16) as primary treatment was conducted. Patients with macroscopic vascular invasion or metastasis were excluded.

### Clinical variables

**Figure 1.**
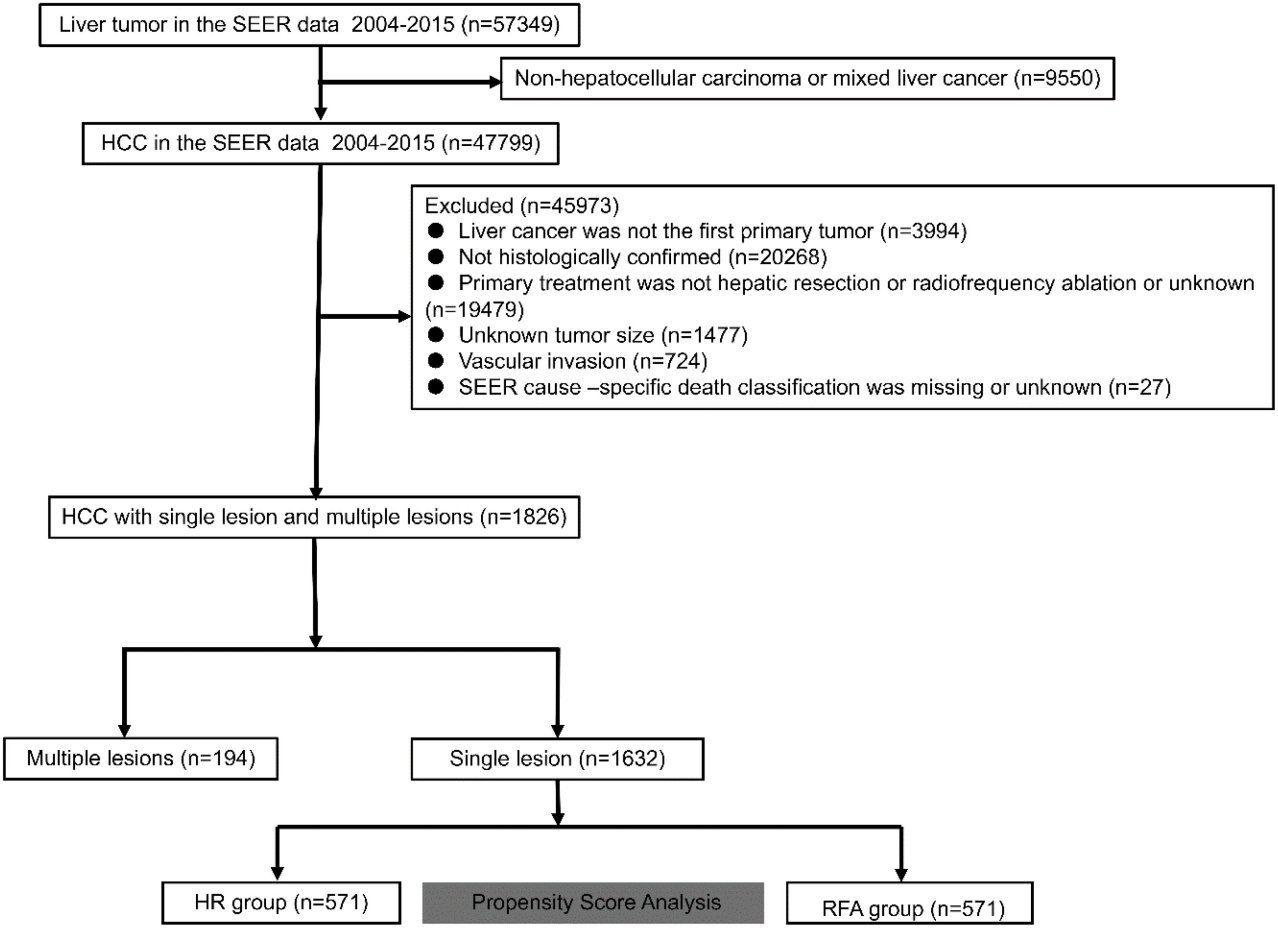
**Flowchart of the screening process of patients with solitary HCC.** HCC: Hepatocellular carcinoma; SEER: Surveillance, Epidemiology, and End Results; RFA: Radiofrequency ablation; HR: Hepatic resection.

Variables, including age, sex, primary tumor size, tumor count, α-fetoprotein (AFP) level, fibrosis score (Ishak score), survival time, SEER cause-specific death classification, and vital status recode (study cut-off used), were extracted from the database. According to tumor count, only HCC cases with single lesion were enrolled into the final analysis in our study. The primary outcome was OS, which is defined in the SEER database at the time until death as a result of any cause. The secondary outcome was DSS, and survival was censored at death from causes other than the primary disease. After the application of the criteria, 1632 patients were enrolled in the analysis. The detailed flowchart demonstrating the screening process is illustrated in Figure 1.

### Subgroup analysis and survival analysis after PSM

A total of 1632 solitary HCC patients were divided into two groups by age: >65 and ≤65. Patients in each age group were divided into three subgroups by tumors size: 0–2, 2–5, and >5 cm. After balancing the baseline characteristics of patients in the RFA group and HR group at each age group, survival analysis was conducted in each subgroup to compare the efficacy of RFA and HR.

### Hierarchical regression analysis

The aim of this study was to investigate the effect of age and tumor size on the efficacy of HR and RFA. To verify the results of survival analysis after PSM, hierarchical regression analysis was performed to determine the influence of other factors on prognosis.

### Ethical statement

We submitted our request to access the SEER database (account no. 19003-Nov2020) to obtain data for our study. The SEER database is shared, and all the patients’ information is accessible; thus, obtaining ethics committee approval or informed consent from the patients was not needed.

### Statistical analysis

The characteristics of patients who received RFA and HR were compared using the chi-squared test for categorical variables and Student’s *t*-test for continuous variables. PSM was carried out to maintain a balance between the RFA and HR groups. We calculated the propensity score using logistic regression with the variables that were potentially associated with DSS and OS or that were unbalanced between the two groups: age, sex, tumor size, AFP level, and fibrosis. Patients were matched using a 1:1 nearest-neighbor approach without replacement. A total of 1142 patients (571 in each group) were selected after matching. We performed univariate analyses for all variables and variables with *p* < 0.05 were included in the multivariate analysis. For multivariate analysis of the matched population, a Cox proportional hazards regression analysis was used to determine the simultaneous impact of potential confounders, including age, AFP level, and fibrosis. Hierarchical regression analysis was performed to verify the results of survival analysis after PSM. The Kaplan–Meier method with a log-rank test was applied to compare the survival of patients. All statistical tests were two-sided, and *p* < 0.05 was considered statistically significant. For all analyses, we calculated hazard ratios with 95% confidence interval (CI). All statistical analyses were performed with SPSS 26.0 (SPSS Inc., Chicago, IL, USA) and R software (version 4.0.4).

## Results

### Baseline characteristics

Baseline characteristics of 1632 patients with solitary HCC before PSM are presented in Table 1. Before PSM, patients in the RFA group had a higher proportion in the 0–2 cm tumor size but had a lower proportion in tumor size >5 cm compared to those in the HR group. Besides, in the younger patient group (age ≤65), patients in the RFA group were significantly older, had a higher level of AFP, and were less likely to be classified as having cirrhosis compared to those in the HR group. The baseline characteristics of patients after PSM (*n* ═ 1142) are presented in Table 2. After PSM, variables in each population stratified by tumor size were approximately balanced between the RFA and HR groups (Table 3 for patients > 65 years old and Table 4 for patients ≤65 years old).

**Table 1:** Baseline characteristics of patients with solitary HCC before PSM

**Characteristics**	**Age > 65**			**Age ≤ 65**			
	**HR (*n* = 403)**	**RFA (*n* = 250)**	***p***	**HR (*n* = 560)**	**RFA (*n* = 419)**	***p***	**DF**
*Age (years)*							
66–75 (<50)	262	152	0.397	135	50	<0.001	2
76–85 (51–65)	132	89		425	369		
>85	9	9					
*Sex*							
Male	281	172	0.803	422	342	0.019	1
Female	122	78		138	77		
*Tumor size*							
≤2 cm	38	51	<0.001	83	114	<0.001	2
2–5 cm	180	184		269	277		
>5 cm	185	15		208	28		
*Fibrosis*							
Severe or cirrhosis	79	56	0.391	414	275	0.005	1
None or not stated	324	194		146	144		
*AFP*							
Elevated	157	117	0.135	272	250	0.001	2
Normal or borderline	153	85		171	115		
Unknown	93	48		117	54		

AFP: α-fetoprotein; DF: Degree of freedom; HCC: Hepatocellular carcinoma; HR: Hepatic resection; PSM: Propensity score matching; RFA: Radiofrequency ablation.

**Table 2:** Baseline characteristics of patients with solitary HCC after PSM

**Characteristics**	**Age > 65**			**Age ≤ 65**			
	**HR (*n* = 223)**	**RFA (*n* = 223)**	***p***	**HR (*n* = 348)**	**RFA (*n* = 348)**	***p***	**DF**
*Age (years)*							
66–75 (<50)	145	148	0.953	47	48	0.912	2
76–85 (51–65)	71	68		301	300		
>85	7	7					
*Sex*							
Male	142	152	0.318	258	283	0.023	1
Female	81	71		90	65		
*Tumor size*							
≤2 cm	38	38	0.983	83	71	0.548	2
2–5 cm	169	170		238	249		
>5 cm	16	15		27	28		
*Fibrosis*							
Severe or cirrhosis	46	49	0.729	229	238	0.468	1
None or not stated	177	174		119	110		
*AFP*							
Elevated	91	101	0.484	181	206	0.102	2
Normal or borderline	87	75		98	91		
Unknown	45	47		69	51		

AFP: α-fetoprotein; DF: Degree of freedom; HCC: Hepatocellular carcinoma; HR: Hepatic resection; PSM: Propensity score matching; RFA: Radiofrequency ablation.

**Table 3:** Characteristics of patients > 65 years old stratified by tumor size after PSM

**Characteristics**	**≤2 cm**			**2–5 cm**			**>5 cm**			
	**RFA**	**HR**	***p***	**RFA**	**HR**	***p***	**RFA**	**HR**	***p***	**DF**
*Age (years)*										
66–75	27	30	0.542	114	114	1.000	7	1	0.019	2
76–85	10	6		51	51		7	14		
>85	1	2		5	4		1	1		
*Sex*										
Male	24	23	0.813	116	118	0.752	12	1	<0.001	1
Female	14	15		54	51		3	15		
*Fibrosis*										
Severe or cirrhosis	31	27	0.280	131	134	0.619	12	16	0.101	1
None or not stated	7	11		39	35		3	0		
*AFP*										
Elevated	18	16	0.614	75	68	0.769	8	7	0.248	2
Normal or borderline	12	16		58	62		5	9		
Unknown	8	6		37	39		2	0		

AFP: α-fetoprotein; DF: Degree of freedom; HR: Hepatic resection; PSM: Propensity score matching; RFA: Radiofrequency ablation.

**Table 4:** Characteristics of patients ≤ 65 years old stratified by tumor size after PSM

**Characteristics**	**≤2 cm**			**2–5 cm**			**>5 cm**			
	**RFA**	**HR**	***p***	**RFA**	**HR**	***p***	**RFA**	**HR**	***p***	**DF**
*Age (years)*										
<50	13	18	0.602	31	23	0.328	4	6	0.503	1
51–65	58	65		218	215		24	21		
*Sex*										
Male	55	62	0.689	203	183	0.207	25	13	0.001	1
Female	16	21		46	55		3	14		
*Fibrosis*										
Severe or cirrhosis	47	57	0.743	171	157	0.524	20	15	0.221	1
None or not stated	24	26		78	81		8	12		
*AFP*										
Elevated	39	46	0.195	153	116	0.019	14	13	0.230	2
Normal or borderline	24	20		57	71		10	7		
Unknown	8	17		39	51		4	1		

AFP: α-fetoprotein; DF: Degree of freedom; HR: Hepatic resection; PSM: Propensity score matching; RFA: Radiofrequency ablation.

**Figure 2.**
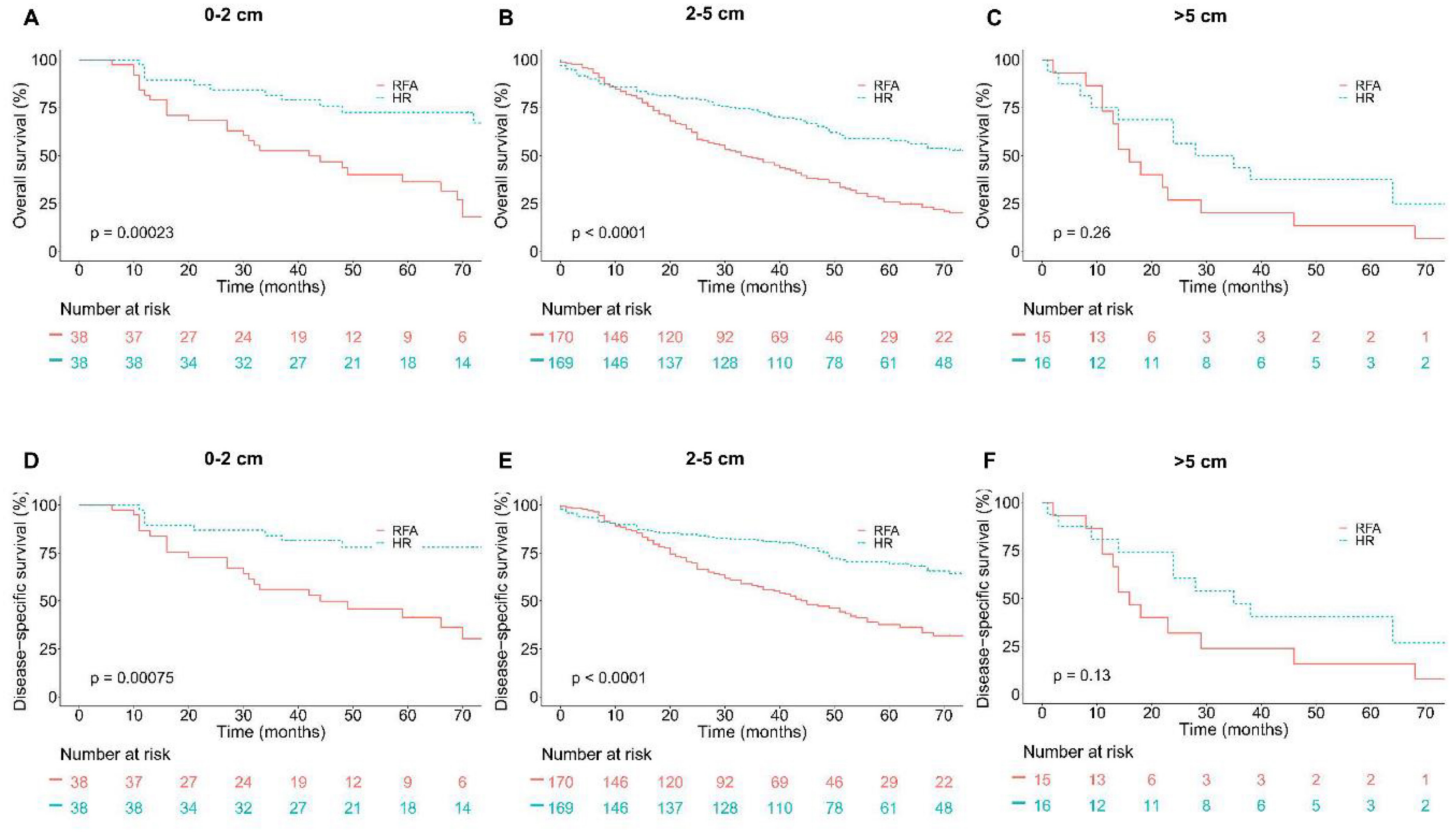
**Overall survival (OS) and Disease-specific survival (DSS) in older group (age > 65) based on tumor size groups and interventions within each group.** (A) OS in 0–2 cm group; (B) OS in 2–5 cm group; (C) OS in over 5 cm group; (D) DSS in 0–2 cm group; (E) DSS in 2–5 cm group; (F) DSS in over 5 cm group.

**Figure 3.**
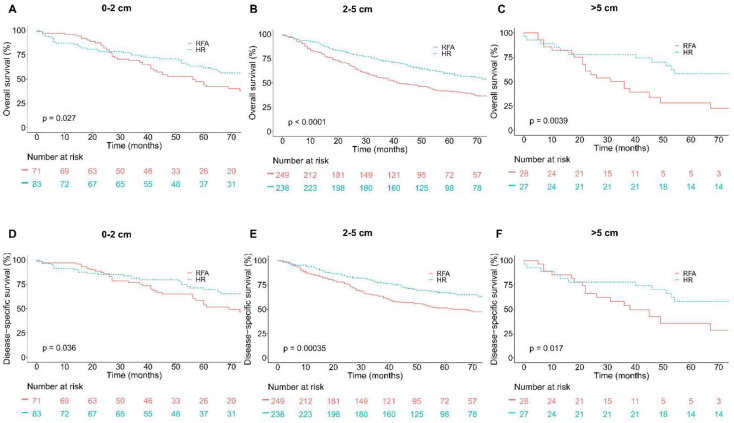
**Overall survival (OS) and disease-specific survival (DSS) analyses in younger group (age ≤ 65) based on tumor size groups and interventions within each group after PSM.** (A) OS in 0–2 cm group; (B) OS in 2–5 cm group; (C) OS in over 5 cm group; (D) DSS in 0–2 cm group; (E) DSS in 2–5 cm group; (F) DSS in over 5 cm group.

### Survival analysis after PSM

After PSM, Kaplan–Meier survival analyses were performed based on tumor size groups (0–2, 2–5, and >5 cm groups) and interventions. In patients >65 years old, as shown in Figure 2, in patients with tumors measuring 0–2 and 2–5 cm, patients in the HR group had a significantly better OS and DSS compared with patients in the RFA group. However, in patients with tumors >5 cm, OS and DSS between the HR group and RFA group showed no significant difference (*p* ═ 0.26 and *p* ═ 0.13, respectively). Likewise, in patients ≤65 years old, HR showed significant efficacy advantage over RFA in patients with tumors measuring 0–2, 2–5, and >5 cm. The results were shown in Figure 3.

OS and DSS between HR and RFA after PSM stratification by tumor size were compared. Results in Table 5 show that the survival rates of patients in the HR group with tumors measuring 0–2 and 2–5 cm were significantly higher than that of patients in the RFA group for elderly patients (age > 65). However, the survival rates of patients with tumors >5 cm did not differ significantly between the HR and RFA groups (*p* ═ 0.262 and *p* ═ 0.129, respectively). Likewise, in patients ≤65 years old, the survival rates of patients in the HR group with tumors measuring 0–2, 2–5, and >5 cm were significantly higher than that of patients in the RFA group.

After adjusting confounding factors affecting OS and DSS, the risk of mortality was compared by calculating the hazard ratio. The results were shown in Table 6. First, we compared the risk of mortality in patients > 65 years old stratification by tumor size. In patients with tumors measuring 0–2 cm, patients in the HR group had a significantly lower risk of mortality compared with patients in the RFA group (OS hazard ratio, 0.31; 95% CI, 0.16–0.60, *p* ═ 0.001 and DSS hazard ratio, 0.30; 95% CI, 0.14–0.63, *p* ═ 0.001). Among those with tumors measuring 2–5 cm, patients in the HR group had also a significantly lower risk of mortality compared with patients in the RFA group (OS hazard ratio, 0.46; 95% CI, 0.35–0.60, *p* < 0.001 and DSS hazard ratio, 0.42; 95% CI, 0.30–0.58, *p* < 0.001). However, in patients with tumors >5 cm, the OS and DSS did not differ significantly between HR and RFA (OS hazard ratio, 0.65; 95% CI, 0.30–1.40, *p* ═ 0.269 and DSS hazard ratio, 0.53; 95% CI, 0.23–1.23, *p* ═ 0.139). Besides, in patients ≤65 years old, after adjusting confounding factors affecting OS and DSS, patients in the HR group had a significantly lower risk of mortality compared with patients in the RFA group in all tumor-size groups.

### Multivariate analysis after PSM

As is portrayed in Table 7, the multivariate analysis of survival in patients with age >65 revealed that the age, tumor size, and type of surgery significantly affected OS. Tumor size, AFP level, and type of surgery also had a significant effect on DSS. As is depicted in Table 8, the multivariate analysis of survival in patients with age ≤65 revealed that the age, AFP level, and type of surgery significantly affected OS. Age and type of surgery also had a significant effect on DSS.

**Table 5:** Survival of patients with solitary HCC by treatment group and tumor size (after PSM)

**Size group, %**	**0–2 cm**			**2–5 cm**			**>5 cm**		
	**RFA, %**	**HR, %**	***p***	**RFA, %**	**HR, %**	***p***	**RFA, %**	**HR, %**	***p***
*For patients aged > 65*									
3-years OS	52.6	78.9	<0.001 (DF=1)	48.2	72.8	<0.001 (DF=1)	13.3	43.8	0.262 (DF=1)
5-years OS	36.2	67.0		25.9	58.0		6.7	25.0	
3-years DSS	55.9	81.4	0.001 (DF=1)	57.3	81.6	<0.001 (DF=1)	16.0	47.1	0.129 (DF=1)
5-years DSS	41.4	71.4		36.2	69.3		8.0	26.9	
*For patients aged ≤65*									
3-years OS	67.6	73.4	0.027 (DF=1)	54.7	72.5	<0.001	42.9	74.1	0.004 (DF=1)
5-years OS	43.8	61.5		41.0	59.3		22.4	58.0	
3-years DSS	75.1	79.7	0.036 (DF=1)	63.3	77.2	<0.001 (DF=1)	53.9	74.1	0.017 (DF=1)
5-years DSS	54.0	69.3		50.5	66.3		28.2	58.0	

DSS: Disease-specific survival; DF: Degree of freedom; HCC: Hepatocellular carcinoma; HR: Hepatic resection; OS: Overall survival; PSM: Propensity score matching; RFA: Radiofrequency ablation.

**Table 6:** Hazard ratios after adjusting confounding factors (after PSM)

**Size group**	**Age > 65**			**Age ≤ 65**		
	**Hazard ratio**	**95% CI**	***p***	**Hazard ratio**	**95% CI**	***p***
*0–2 cm*						
HR vs RFA (OS)	0.31	0.16–0.60	**<0.001** (DF=1)	0.61	0.39–0.95	**0.029** (DF=1)
HR vs RFA (DSS)	0.30	0.14–0.63	**0.001** (DF=1)	0.58	0.35–0.97	**0.039** (DF=1)
*2–5 cm*						
HR vs RFA (OS)	0.46	0.35–0.60	**<0.001** (DF=1)	0.59	0.46–0.75	**<0.001** (DF=1)
HR vs RFA (DSS)	0.42	0.30–0.58	**<0.001** (DF=1)	0.60	0.46–0.80	**<0.001** (DF=1)
*>5 cm*						
HR vs RFA (OS)	0.65	0.30–1.40	0.269 (DF=1)	0.36	0.18–0.74	**0.005** (DF=1)
HR vs RFA (DSS)	0.53	0.23–1.23	0.139 (DF=1)	0.41	0.19–0.87	**0.021** (DF=1)

CI: Confidence interval; DSS: Disease-specific survival; DF: Degree of freedom; HR: Hepatic resection; OS: Overall survival; PSM: Propensity score matching; RFA: Radiofrequency ablation. Bold type indicates statistical significance.

**Table 7:** Multivariate analysis for survival in patients with age >65

**Variable**	**Overall survival**			**Disease-specific survival**		
	**Hazard ratio**	**95% CI**	***p***	**Hazard ratio**	**95% CI**	***p***
*Tumor size*						
≤2 cm	Reference			Reference		
2–5 cm	1.33	0.96–1.85	0.092 (DF=2)	1.21	0.84–1.76	0.312 (DF=2)
>5 cm	2.49	1.52–4.09	**<0.001** (DF=2)	2.83	1.66–4.83	**<0.001** (DF=2)
*Type of surgery*						
RFA	Reference			Reference		
HR	0.44	0.35–0.56	**<0.001** (DF=1)	0.43	0.32–0.56	**<0.001** (DF=1)
*AFP*						
Elevated	Reference			Reference		
Normal or borderline				0.70	0.51–0.96	**0.027** (DF=2)
Unknown				0.89	0.62–1.26	0.503 (DF=2)

Bold type indicates statistical significance. AFP: α-fetoprotein; CI: Confidence interval; DF: Degree of freedom; HR: Hepatic resection; RFA: Radiofrequency ablation.

**Table 8:** Multivariate analysis for survival in patients with age ≤ 65

**Variable**	**Overall survival**			**Disease-specific survival**		
	**Hazard ratio**	**95% CI**	***p***	**Hazard ratio**	**95% CI**	***p***
*Age (years)*						
<50	Reference			Reference		
51–65	1.84	1.32–2.56	**< 0.001** (DF=1)	1.54	1.07–2.22	**0.019** (DF=1)
*Type of surgery*						
RFA	Reference			Reference		
HR	0.56	0.46–0.69	**< 0.001** (DF=1)	0.57	0.45–0.73	**<0.001** (DF=1)
*AFP*						
Elevated	Reference			Reference		
Normal or borderline	0.71	0.56–0.91	**0.006** (DF=2)			
Unknown	0.98	0.75–1.28	0.863 (DF=2)			

AFP: α-fetoprotein; CI: Confidence interval; DF: Degree of freedom; HR: Hepatic resection; RFA: Radiofrequency ablation. Bold indicates statistical significance.

### Hierarchical regression analysis and survival before PSM

**Figure S1.**
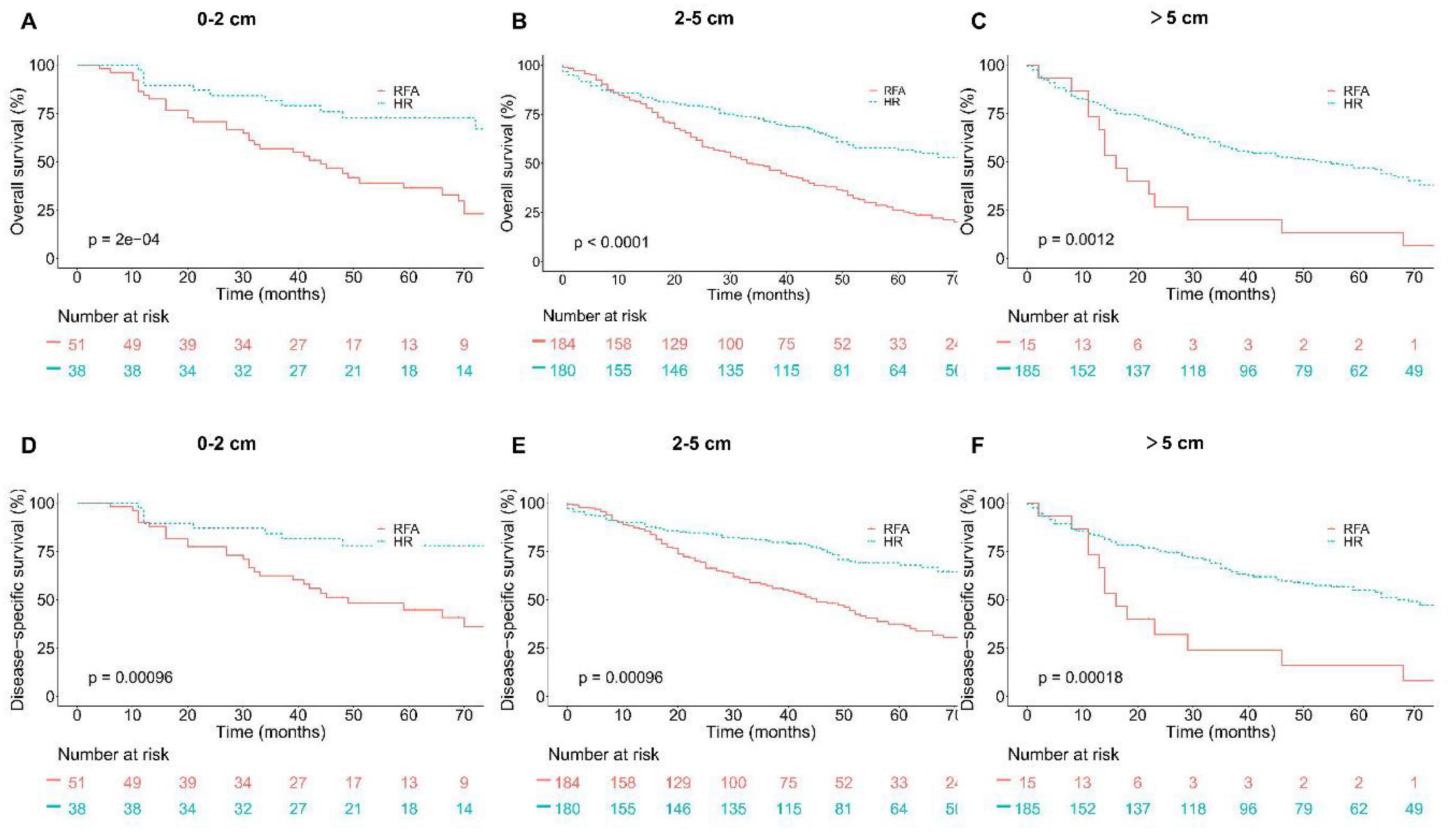
**Overall survival (OS) and disease-specific survival (DSS) in older group (age>65) based on tumor size groups and interventions within each group before PSM.** (A) OS in 0–2 cm group; (B) OS in 2–5 cm group; (C) OS in over 5 cm group; (D) DSS in 0–2 cm group; (E) DSS in 2–5 cm group; (F) DSS in over 5 cm group.

**Figure S2.**
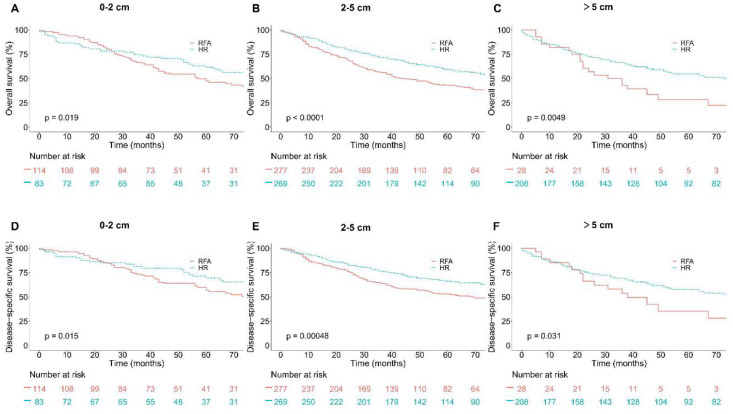
**Overall survival (OS) and disease-specific survival (DSS) analyses in younger group (age **≤** 65) based on tumor size groups and interventions within each group before PSM.** (A) OS in 0–2 cm group; (B) OS in 2–5 cm group; (C) OS in over 5 cm group; (D) DSS in 0–2 cm group; (E) DSS in 2–5 cm group; (F) DSS in over 5 cm group.

The results of hierarchical regression analysis in Table S1 showed that other variables (sex, AFP, and fibrosis) had no significant impact on prognosis after stratification according to the treatment mode. Before PSM, the results of survival analysis showed that OS and DSS were significantly better with HR than those with RFA in patients ≤65 years old regardless of tumor size and in patients >65 years old with tumors ≤5 cm, which was consistent with that after PSM. However, for patients>65 years old with tumors >5 cm, HR was significantly superior to RFA on OS and DSS, which was inconsistent with the results after PSM. See Figures S1 and S2 for the results.

## Discussion

With the continuous progress of ablation techniques, several ablation technologies, including RFA, microwave ablation, percutaneous ethanol injection, and cryoablation, have been used for the treatment of HCC. Currently, RFA is the most well studied and most widely used ablative method due to its proven efficacy and safety [31]. RFA has become the standard of care for unresectable early HCC and has even been found to be competitive with surgery in cases of a single tumor less than 3 cm [32, 33]. For solitary HCC with tumor ≤2 cm in size, RFA can be performed as a first-line treatment and is more cost-effective than HR [34]. However, the better treatment choice in this population remains controversial. One study showed that the efficacy of RFA was still inferior to that of HR in HCCs smaller than 2 cm because of worse survival outcomes and higher recurrence rates [35]. In a study conducted by Jiang et al., the efficacy of RFA and HR was compared in elderly HCC patients (age > 65) with tumors smaller than 2 cm, and the results showed that RFA and HR had similar OS and DSS. The author concluded that RFA should be performed rather than HR considering that RFA is more cost-effective than HR and less influenced by age [36]. The advent of novel techniques has largely improved the efficacy of ablation. Now RFA can be applied to tumors up to 5 cm with similar OS and DFS in comparison to HR [37]. As the diameter of a single tumor increases, it is more difficult to achieve complete ablation, which is the main reason for tumor recurrence and progression after RFA [38]. Meta-analysis of 95 studies, including 5224 liver tumors treated by RFA reported a local recurrence rate of 12.4%. Local recurrence was substantially higher following treatment of tumors >3–5 cm (24.1%) or >5 cm (58.1%) in diameter [39]. In addition to tumor size, other prognosis biomarkers have been found for liver malignancies after RFA, such as lymphocyte-to-monocyte ratio, AFP, and hyperglycemia [40–42].

Despite the fact that HR remains the first-line treatment option with a 5-year survival rate of > 50% for patients with good hepatic functional reserve and a low operative mortality, this treatment is subject to underlying liver cirrhosis or multiple lesions [43–46]. Most patients with HCC are diagnosed with cirrhosis, and the degree of fibrotic burden within cirrhosis is significantly related to late recurrence after resection [47]. To decrease the possibility of liver failure after hepatectomy, the future liver remnant should be at least one-third of the total liver volume and 40%–50% in patients with parenchymal liver disease [48, 49].

Elderly patients generally have a high incidence of comorbid diseases and are usually divided into high-risk group for surgical resection [50, 51]. Hence, RFA might be a potentially advantageous treatment option for elderly patients in terms of its less morbidity and high quality of life [52, 53]. It has been controversial whether elderly patients with HCC would benefit more from RFA or HR. In one study, Peng et al. [54] concluded that RFA had better efficacy than HR for elderly patients with HCC tumors <3 cm. In a large nationwide study by Kaibori et al. [55], the efficacy of RFA and HR was compared in patients aged ≥75 years old, and the author concluded that the elderly patients (aged ≥75) had significantly better recurrence-free survival after HR for HCC than after RFA and that HR decreased the risk of tumor recurrence and improved OS in patients aged ≥75 years with primary HCC tumors of ≤3 cm in diameter. Given these facts, our study aimed to address the issue that whether RFA or HR should be performed in elderly patients with a single tumor nodule based on the SEER database.

To investigate the influence of age and tumor size on the treatment choice of patients with solitary HCC, a subgroup analysis was conducted. Patients after PSM were divided into two subgroups by age as follows: ≤65 and >65. Then each age group was divided into three subgroups according to the tumor size as follows: (1) 0–2 cm, (2) 2–5 cm, and (3) >5 cm. Finally, the effects of RFA and HR on survival were compared in each subgroup. In our study, we defined ≥65 years of age as elderly because a British study reported that HCC patients aged ≥65 received less or less-active treatment and had poorer survival than younger individuals [56]. The cut-off value of 2 cm was based on the studies showed that RFA was a more appropriate treatment for patients with a single tumor <2 cm [20, 21]. However, a recent study also concluded that HR provided better survival than RFA in this population [57]. Concerning tumors measuring smaller than 5 cm, a randomized-controlled trial performed by Chen et al. [24] showed for the time that RFA and surgical resection demonstrated indistinguishable efficacy for patients with a single tumor ≤5 cm. However, another study conducted by Huang et al. [58], in patients within Milan criteria discovered that hepatectomy was superior to RFA in terms of better survival and lower tumor recurrence rates.

In our study, we found that OS and DSS differ significantly between the RFA and HR groups among elderly patients with tumors ≤5 cm. Specifically, HR provided better OS and DSS with statistical significance compared with RFA in patients with tumor size ≤5 cm. Notably, in patients with tumors measuring 0–2 cm, we found that HR was associated with better OS and DSS compared with RFA; this finding was different from the results of studies by Peng et al. [54] and Jiang et al. [36]. This discrepancy might be partially explained by the following two aspects. First, the relatively small sample size in the study by Peng et al. (*n* ═ 63 in the RFA group and *n* ═ 60 in the HR group). Second, patients included in the study by Jiang et al. were not limited to those with a single tumor lesion. The latest version of BCLC guidelines demonstrates that due to the high risk of recurrence, the potential for LT should be considered; for HCC patients with single tumor ≤2 cm, if they are not candidates for LT, ablation is the first choice, and if they are candidates and meet the resection conditions, resection is the first choice [59]. The purpose of resection is to identify predictors of tumor recurrence, such as microvascular invasion and tumor satellite and, finally, consider LT due to such risk. However, worldwide, due to the shortage of donor liver and the restriction of recruitment policy, transplantation is rarely carried out. From the perspective of economy and security, RFA is preferred over HR in solitary HCC with tumors ≤2 cm [34]. In this study, however, our view is that regardless of whether the patient is a candidate for LT or not, HR rather than RFA should be given priority, because HR has more survival advantages in OS and DSS. Moreover, pathological examination of resected liver tissue could identify high-risk factors for recurrence, such as microvascular invasion and satellite [60–62]. If those risk factors are present, LT should be reconsidered.

In elderly patients with tumors >5 cm, we found for the first time that OS and DSS did not differ significantly between the RFA and HR groups. The survival difference between the two groups was not statistically significant but the HR group had a trend toward better survival outcomes compared with the RFA group.

When the diameter of a single tumor is more than 5 cm, it is not beneficial for patients to accept resection or RFA alone due to high recurrence rate and poor survival [26, 39].

In a study by Duan et al., the efficacy of combined transcatheter arterial chemoembolization (TACE) and RFA for large HCC (≥8 cm) was evaluated, and the results showed that this combination treatment led to synergistic effects of ablation and chemotherapy, which improved the efficacy of ablation, reduced tumor recurrence, and prolonged survival [63]. Moreover, TACE with RFA is superior to hepatectomy in HCC patients beyond the Milan criteria because the combination treatment has advantages over survival rates and median survival time [64]. Besides, the sample size of the group with tumors >5 cm over 65 years old was too small after PSM. Therefore, treatment recommendation could not be provided for this population in our study, more evidence is needed to address this issue.

In patients ≤65 years old with tumors of any size, we found that HR provided better OS and DSS with statistical significance compared with RFA. As a result, in patients ≤65 years old, HR is recommended for solitary HCC patients with sufficient liver function.

In order to verify the reliability of the results of survival analysis after PSM, we conducted hierarchical regression analysis. The results showed that factors other than age and tumor size did not significantly affect the prognosis after stratification according to treatment methods. We infer that the difference in survival is more likely due to different treatment methods after grouping according to age and tumor size. In addition, for patients with tumors ≤5 cm regardless of age, the results of survival analysis before and after PSM are consistent. However, for elderly patients with tumors >5 cm, the results of survival analysis before PSM show that HR has survival advantages over RFA. This change may be due to the increase in sample size. Overall, our conclusions are reliable.

There were some limitations in our study. First, some important variables were not available in the SEER database. For example, the location of the tumor, ECOG PS, degree of portal hypertension, comorbidity, liver function, and tumor progression were not recorded in the SEER database. The extent of fibrosis (fibrosis score) was recorded in the SEER database; this factor is relevant to liver function and was kept balanced between the RFA and HR groups after PSM. To some extent, liver function in both groups can be considered balanced. Second, information on details about ablation procedures, such as the frequency used for ablation, temperature achieved in the tumor, complications after RFA, cycles of RFA, and types of liver resection, such as standard hepatectomy, segmentectomy or non-anatomical resection are not available in the SEER database. Third, information about HCC patients treated with TACE combined with RFA are also not available in the SEER database. Despite these limitations, our study was based on a large sample of HCC patients with a single lesion among the whole United States population, making our conclusions convincing.

## Conclusion

For patients with resectable solitary HCC, regardless of age, HR is the better choice not only for tumors ≤2 cm but also for tumors 2–5 cm. For resectable solitary HCC with tumors >5 cm, HR is the better choice for patients ≤65 but for patients >65, the issue of treatment choice needs to be further studied.

## Supplemental Data

**Table S1:** Hierarchical regression analysis in 1632 solitary HCC patients

**Variable**	**Overall survival**			**Disease- specific survival**		
	**Hazard ratio**	**95% CI**	***p***	**Hazard ratio**	**95% CI**	***p***
*Age > 65*						
Sex	1.02	0.83-1.26	0.833	1.11	0.88-1.41	0.372
AFP	0.97	0.86-1.11	0.686	0.93	0.80-1.07	0.308
Fibrosis	0.95	0.75-1.21	0.686	0.90	0.68-1.20	0.482
*Age ≤ 65*						
Sex	0.92	0.74-1.13	0.405	0.86	0.68-1.09	0.211
AFP	1.87	0.77-0.98	0.024	0.84	0.74-0.96	0.013
Fibrosis	1.03	0.86-1.24	0.741	0.96	0.78-1.19	0.722
*Tumor size ≤ 2 cm*						
Sex	1.29	0.92-1.81	0.137	1.03	0.69-1.55	0.875
AFP	0.95	0.76-1.18	0.614	0.91	0.71-1.18	0.485
Fibrosis	0.92	0.65-1.29	0.628	0.89	0.60-1.32	0.562
*Tumor size 2-5 cm*						
Sex	1.01	0.83-1.23	0.908	1.01	0.80-1.27	0.919
AFP	0.92	0.82-1.04	0.168	0.87	0.76-1.00	0.052
Fibrosis	1.05	0.88-1.27	0.580	1.03	0.83-1.27	0.818
*Tumor size > 5 cm*						
Sex	0.87	0.66-1.15	0.331	1.02	0.76-1.38	0.888
AFP	0.92	0.78-1.08	0.316	0.87	0.73-1.05	0.146
Fibrosis	0.90	0.64-1.27	0.541	0.80	0.54-1.18	0.263
